# Embracing dynamic public health policy impacts in infectious diseases responses: leveraging implementation science to improve practice

**DOI:** 10.3389/fpubh.2023.1207679

**Published:** 2023-08-17

**Authors:** Westyn Branch-Elliman, A. Rani Elwy, David A. Chambers

**Affiliations:** ^1^VA Boston Healthcare System, Department of Medicine, Section of Infectious Diseases, Boston, MA, United States; ^2^VA Center for Healthcare Organization and Implementation Research (CHOIR), Boston, MA, United States; ^3^Harvard Medical School, Boston, MA, United States; ^4^Department of Psychiatry and Human Behavior, Warren Alpert Medical School, Brown University, Providence, RI, United States; ^5^Division of Cancer Control and Population Sciences, National Cancer Institute, National Institutes of Health, Rockville, MD, United States

**Keywords:** pandemic response, public policy, implementation science, non-pharmaceutical interventions, dynamic sustainability framework, infectious diseases, COVID-19

## Abstract

**Rationale:**

The host-pathogen relationship is inherently dynamic and constantly evolving. Applying an implementation science lens to policy evaluation suggests that policy impacts are variable depending upon key implementation outcomes (feasibility, acceptability, appropriateness costs) and conditions and contexts.

**COVID-19 case study:**

Experiences with non-pharmaceutical interventions (NPIs) including masking, testing, and social distancing/business and school closures during the COVID-19 pandemic response highlight the importance of considering public health policy impacts through an implementation science lens of constantly evolving contexts, conditions, evidence, and public perceptions. As implementation outcomes (feasibility, acceptability) changed, the effectiveness of these interventions changed thereby altering public health policy impact. Sustainment of behavioral change may be a key factor determining the duration of effectiveness and ultimate impact of pandemic policy recommendations, particularly for interventions that require ongoing compliance at the level of the individual.

**Practical framework for assessing and evaluating pandemic policy:**

Updating public health policy recommendations as more data and alternative interventions become available is the evidence-based policy approach and grounded in principles of implementation science and dynamic sustainability. Achieving the ideal of real-time policy updates requires improvements in public health data collection and analysis infrastructure and a shift in public health messaging to incorporate uncertainty and the necessity of ongoing changes. In this review, the Dynamic Infectious Diseases Public Health Response Framework is presented as a model with a practical tool for iteratively incorporating implementation outcomes into public health policy design with the aim of sustaining benefits and identifying when policies are no longer functioning as intended and need to be adapted or de-implemented.

**Conclusions and implications:**

Real-time decision making requires sensitivity to conditions on the ground and adaptation of interventions at all levels. When asking about the public health effectiveness and impact of non-pharmaceutical interventions, the focus should be on *when, how*, and *for how long* they can achieve public health impact. In the future, rather than focusing on models of public health intervention effectiveness that assume static impacts, policy impacts should be considered as dynamic with ongoing re-evaluation as conditions change to meet the ongoing needs of the ultimate end-user of the intervention: the public.

## Background

The discovery and subsequent administration of penicillin in 1943 was a major milestone in clinical medicine, saving countless lives (1). However, even before the drug was approved for clinical use, the first reports of antimicrobial resistance were described. Less than 20 years after initial approval, more than 80% of *Staphylococcus aureus* strains were penicillin-resistant (2). In the decades since, a similar story has been described for every antibiotic brought to market. Host-pathogen relationships are inherently dynamic: as hosts develop ways to combat an infectious diseases threat— whether through immunity or treatment—pathogens evolve to evade our advancements.

In addition to an inherently evolving host-pathogen interaction, many factors impacting this interaction are also constantly changing and need to be considered, measured, and integrated into public health policy making. Factors that change over time and therefore determine public policy impact include resource availability, case fatality rate, understanding about modes of transmission, human behaviors, societal expectations, the evidence basis for treatment and prevention and therefore our understanding about the disease, among others.

Maximizing public health policy impact for combating infectious diseases threats necessitates that all of these dynamic factors be measured and evaluated in real-time to continually adapt response plans and achieve maximum public health benefit. Updating policies and recommendations to elevate some interventions and de-escalate others as contextual factors continually evolve is the best evidence-based policy strategy (3, 4). Achieving this ideal requires re-imaging infectious diseases public health policy making as a dynamic and constantly evolving process with the anticipation of change inherently built into health communications and public expectations (5–7). Infrastructure that can support integration of point-of-care data with emerging and evolving evidence and public input to assess ongoing feasibility, acceptability, appropriateness, and costs are needed to achieve safe, efficient and higher quality care and policies (8).

Viewing pandemic responses and the expected impacts of public health policy through the lens of implementation science would enhance emergency preparedness for future pandemics and ultimately improve public health policy impact. The objectives of this review are to: (1) discuss the pipeline from clinical effectiveness to implementation outcomes to ultimate public health impact and introduce the concept of dynamic policy effectiveness (9), (2) to present the COVID-19 pandemic and public health policy as a case study for considering the dynamic host, pathogen, contextual, and evidence changes that evolved over the course of the world-wide emergency responses, and (3) to propose future innovations to support a real-time, learning public health infrastructure that is more adaptable based on changing conditions, context, and evidence. A practical tool for operationalizing the Dynamic Infectious Diseases Public Health Response Framework is presented. The tool is designed to facilitate integration of key implementation outcomes and considerations into infectious diseases response planning (Supplementary material).

## Clinical efficacy and effectiveness vs. public health policy effectiveness and impact

**Figure 1:**
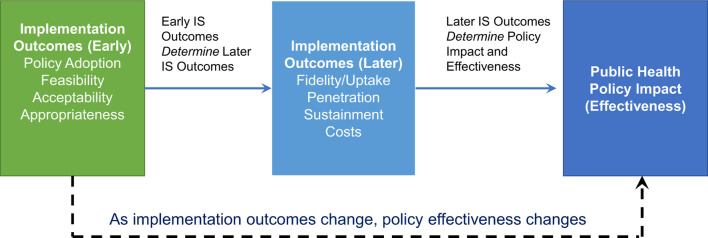
Interplay between implementation outcomes and public health policy impact and effectiveness. Early implementation outcomes (adoption, feasibility, acceptability, appropriateness) have direct impacts on later implementation outcomes, which in turn determine the effectiveness of a public health policy. During a pandemic response, implementation outcomes change, and these changes also alter the expected benefits of public health policy interventions.

Traditional clinical trials evaluate efficacy and effectiveness of an intervention in a controlled setting, whereas implementation trials evaluate how to promote uptake and how to translate potential benefits into tangible improvements (9). Public health policy impact is a downstream consequence of the potential efficacy of the intervention as well as its real-world implementation (Figure 1). The Dynamic Sustainability Framework (DSF) (10) highlights the importance of adapting interventions on *individual* and *systems* levels to maintain and maximize longitudinal impacts in the setting of constantly changing evidence and contexts.

DSF principles also apply to public health systems and public health policy impact. Public health policies are typically composed of a bundle of multiple interventions. To achieve sustained public health impact, public health policies and their component interventions (whether targeted at an individual, organization or society) must adjust and adapt to changing contexts and evidence. In other words, public health policies must be viewed as having *dynamic* public health impacts and *dynamic* effectiveness; a constant level of public health impact while other changes in the system are occurring cannot be assumed.

## Implementation outcomes

Efficacy and effectiveness are the traditional measures for evaluating clinical and public health policies and interventions in controlled research studies. These measures focus on the absolute and relative differences in outcomes among exposed and unexposed groups and are generally conceived of as *static* estimates; in other words, the relative risk reduction associated with receipt of a particular medical intervention is assumed to be *constant* over time.

Implementation science focuses on different outcomes (Table 1) (11). Early implementation outcomes include acceptability, appropriateness, feasibility/availability, and adoption, which is defined as the decision to recommend a specific public health intervention (in contrast with uptake, which is the actual use of the intervention). Later implementation outcomes include fidelity, penetration, and costs. The longest-term implementation outcome is *sustainability*. In implementation science, both *sustainability* and *sustainment* are key terms, with sustainability referring to a property/characteristic of an intervention related to its likely long-term usability whereas sustainment refers to the outcome of whether an intervention was used over a long period of time.

**Table 1:** Implementation outcomes definitions and impact in the setting of constant change.

Implementation outcome^*^	**Definition**	**Examples and key longitudinal changes**
Acceptability	Perception among interested parties that a policy or practice is agreeable, palatable, or satisfactory	*Business and school closures, social distancing* Attitudes about interventions that limited person-to-person contacts changed substantially and rapidly over time. Very-short term viability, and substantial pressure from the community to limit or refuse these types of mitigation interventions.
Appropriateness	The perceived fit, relevance, or compatibility of the policy or practice for a given context^**^	*Hospital Admission Surveillance Testing* Initially, given reports of asymptomatic spread, universal hospital admission screening was considered an appropriate means for limiting risk to patients and staff. Over time, as vaccines became available and downsides of the testing program emerged (e.g., identification of false positives, changing views of the role of asymptomatic spread in driving transmission, concerns about impacts of delayed medical care), the perceived appropriateness of the intervention changed.
Adoption	Decision to recommend the intervention or public health policy	*Masking* The decision at a local, state, or national level to officially recommend a specific masking policy, such as a recommendation or a requirement to wear masks in all indoor areas.
Feasibility	The extent to which a policy or practice can be used within a specific context. Closely related to and overlapping with resource *availability*, which has been proposed as an implementation outcome in the context of vaccination. (12)	*Testing Strategies* Early in the pandemic, testing was not feasible due to limited testing resources. Then, as resource limitations decreased, testing of exposed individuals became a more feasible option for limiting time in quarantine. Then, high rates of spread during the omicron wave made contact tracing infeasible. As individual testing became less feasible, alternative surveillance strategies, such as community wastewater testing, became more widely available and thus a feasible alternative.
Fidelity	The degree to which a policy or practice is implemented as planned. For multifaceted interventions, can be considered as the “dose.”	*Masking* Masking policy recommendations do not necessarily translate into adherence. Fidelity refers to the rate of adherence, which changed longitudinally with availability of other mitigation measures, and changes to acceptability and appropriateness over time.
Penetration	The reach of the policy or practice (e.g., how many people received the intervention/total number of eligible individuals)	*School-based testing programs* School-based testing programs, such as the test-to-stay modified quarantine program, allowed exposed individuals to continue participation in in-person learning. Penetration, or reach, refers to the number of students and schools who are able to participate in the testing program, and is a function of program availability (access) and participation (e.g, consent)
Cost	The cost or impact of the implementation effort (includes intervention costs, costs of implementation, settings)	*Business and school closures, social distancing* The very short-term (i.e., days to weeks) costs and harms of closures are substantial, and increase as duration and extent of closures increases.

* Sustainability is not listed, as the focus of the framework is on using different contextual factors to predict sustainability.

** Context can be defined broadly, and can refer to practice settings, political settings, longitudinal changers, or other factors that impact the perceived fit of a practice or policy.

## Implementation outcomes: the causal pathway to public health impact

**Figure 2:**
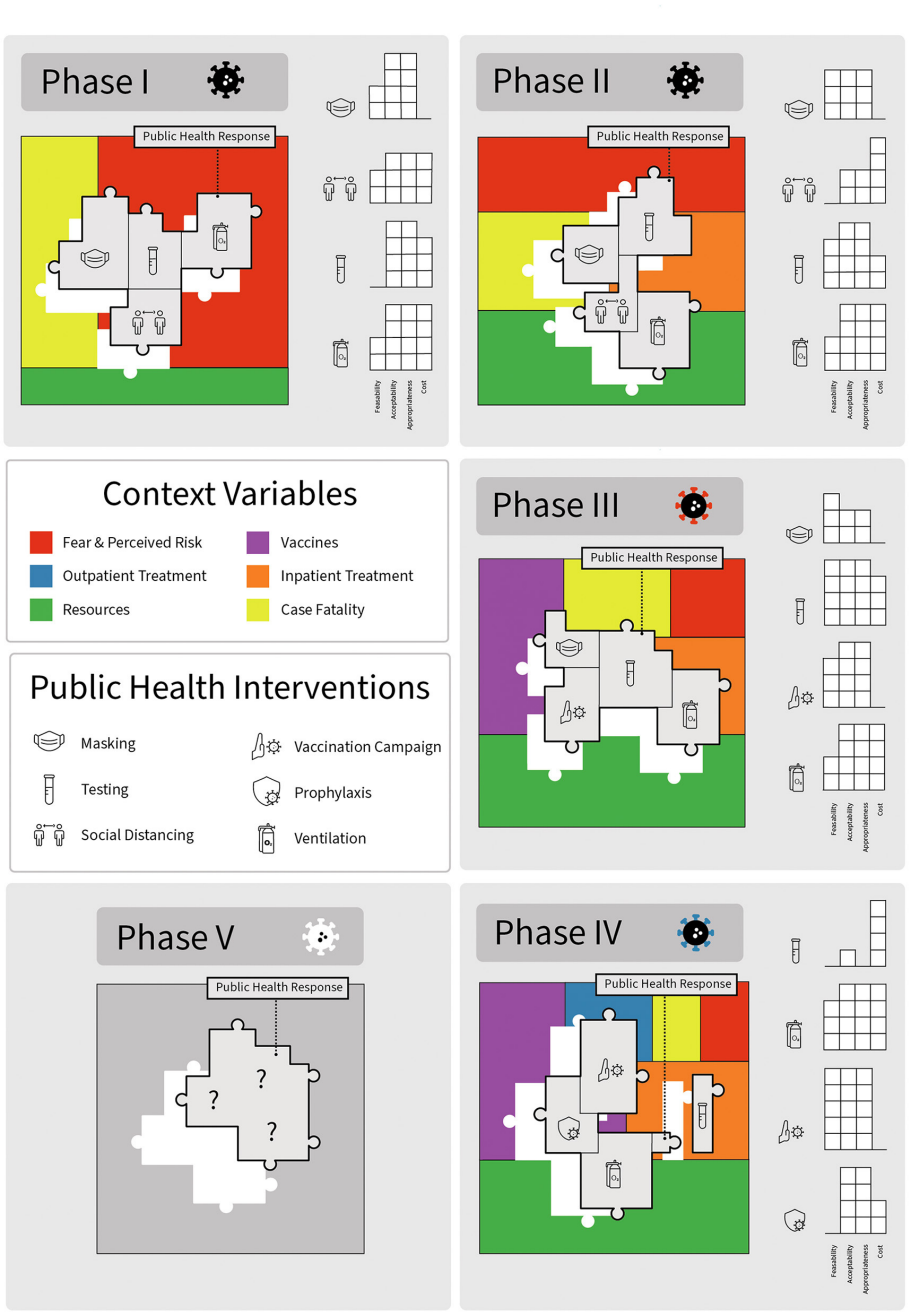
Impact of specific public health policy interventions during different phases of the COVID-19 pandemic: changing context and implementation outcomes alter effectiveness. Each box represents a different phase of the pandemic. Contextual factors are indicated by different colors. Public health policy response interventions are puzzle pieces indicated with relevant icons. Graphs represent changing early implementation outcomes as a function of pandemic phase. The viral icon adjacent to the pandemic phase indicates the circulating variant, which also changed over time.

**Figure 3:**
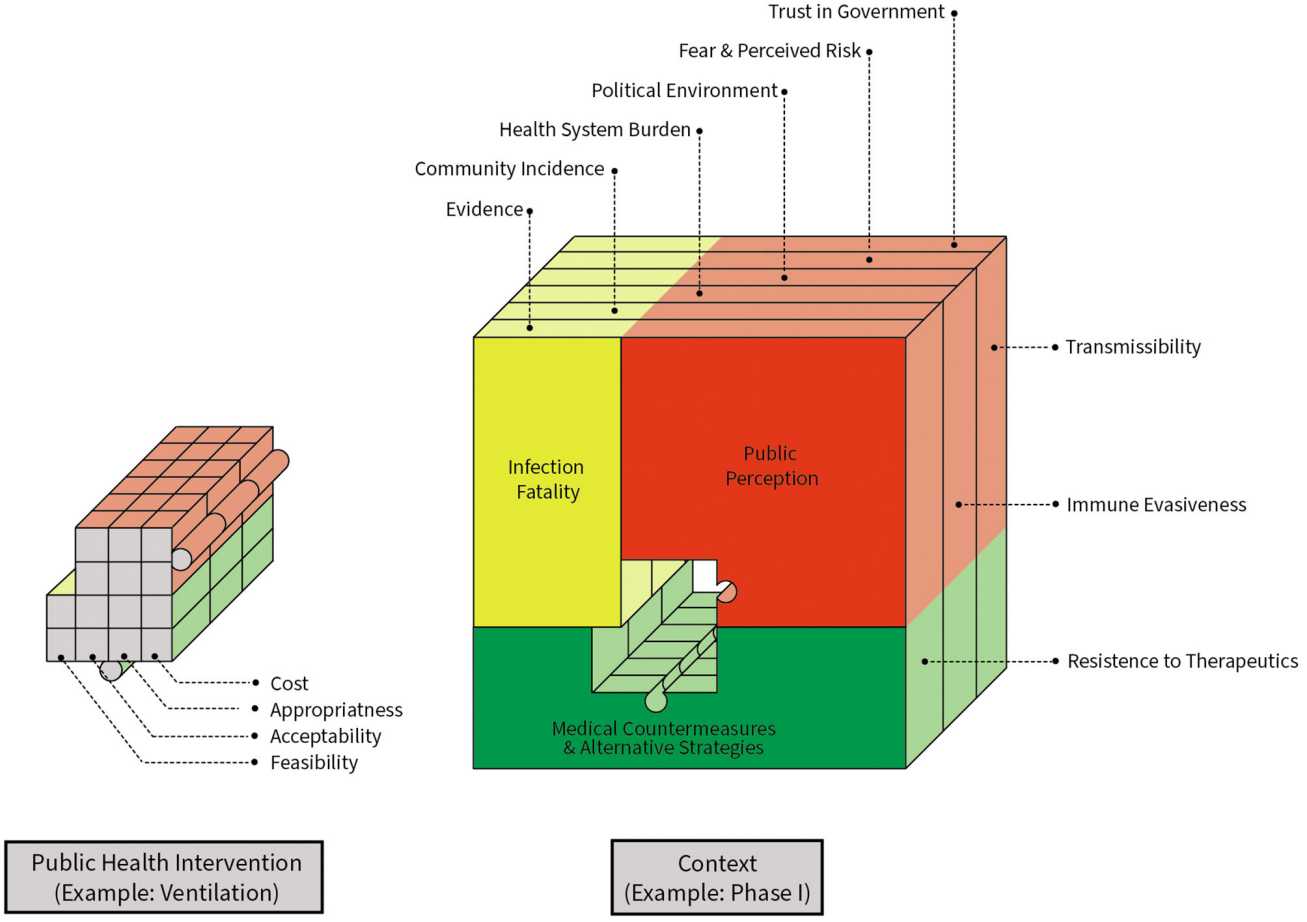
Changing contexts and implementation outcomes alter longitudinal impacts of non-pharmaceutical interventions. The puzzle piece represents a non-pharmaceutical intervention and its expected policy effectiveness in the setting of constant change in multiple dimensions. Key considerations for considering ongoing policy effectiveness are highlighted, including contextual factors and pathogen characteristics.

Importantly, while often not considered when evaluating clinical efficacy and effectiveness, implementation outcomes *directly* impact the expected benefit and impact of public health policies and their components (the individual interventions) (Figure 1). Interventions that are promising in laboratory settings or in idealized clinical trial settings have limited or no impact on public and population health if they are infeasible/unavailable, unacceptable, and/or perceived to be inappropriate by end-users. Further complicating longitudinal evaluations of public health impact and ongoing recommendations, these implementation outcomes themselves are not static – feasibility, which is related to availability (12), acceptability, appropriateness, and costs all vary according to contexts, evolving evidence, resource availability, progress, available alternatives and perceived benefits (Figures 2, 3). Sustainability is a perennial challenge in implementation, particularly if day-to-day behavior change is required and if the intervention is perceived to have substantial downsides. Thus, implementation outcomes are key determinants of public health policy and intervention effectiveness and impact. Implementation outcomes are *also* constantly evolving.

## The COVID-19 pandemic public health response: a case study in constant change

**Figure 4:**
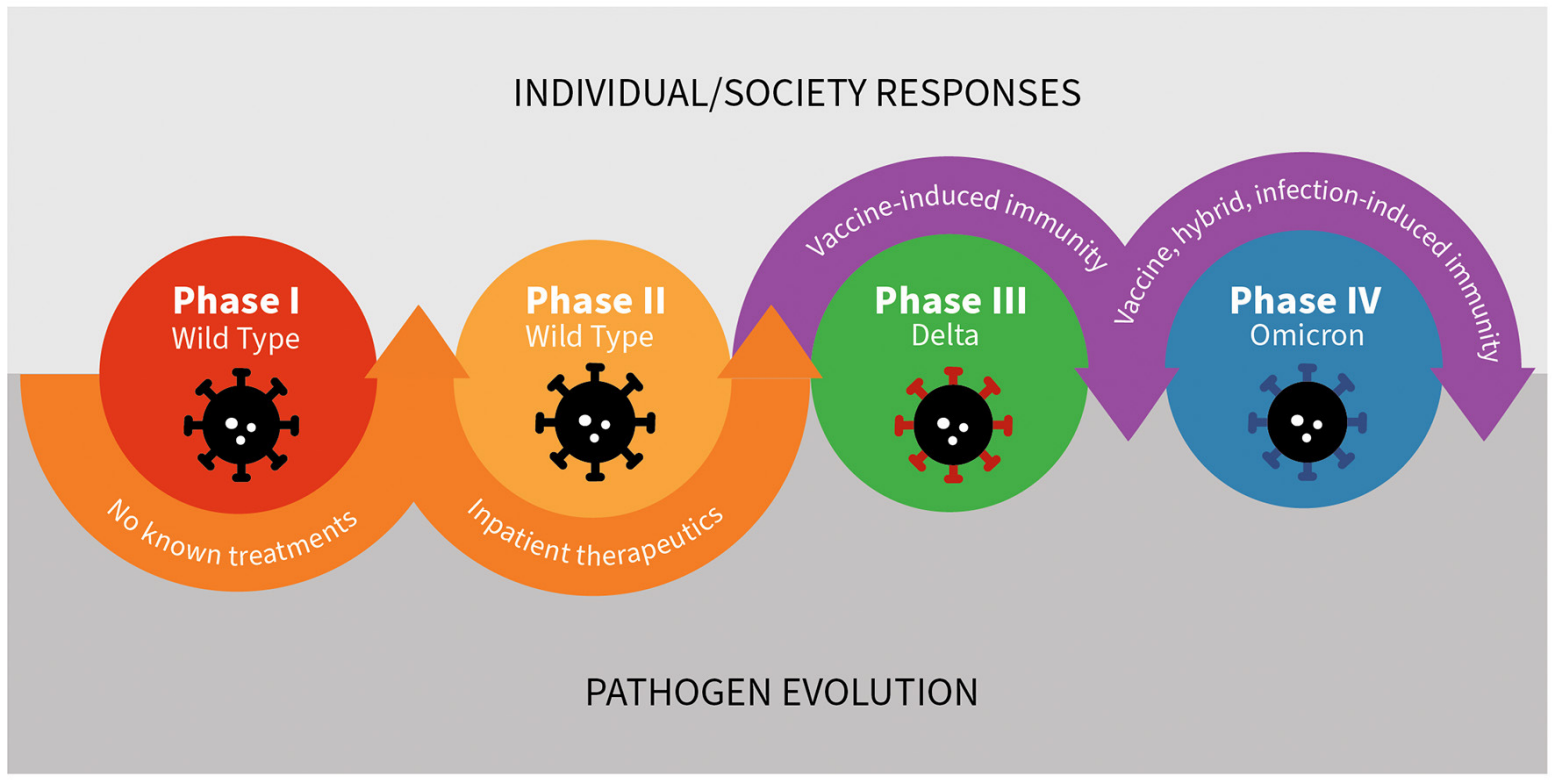
The inherently dynamic nature of the host – pathogen interaction: the case of SARS-CoV-2 and human responses.

Consideration of *dynamic* public health policy effectiveness and impact is particularly important for developing and adapting responses to infectious disease threats. As humans make advancements, such as the development of therapeutics or vaccines, the pathogen evolves in response to human progress (Figure 4). For example, delta and omicron variants both emerged in part due to pressure from vaccine and infection-induced immunity. Mutations arose that rendered once highly effective monoclonal antibody therapies for early treatment and prophylaxis obsolete. Antimicrobial resistance, another critical public health threat in infectious diseases, is a direct downstream consequence of pathogen evolution in response to human innovation. Antimicrobial resistance highlights the generalizability of the dynamic nature of the management and containment of infectious diseases beyond the COVID-19 pandemic.

### The phases of the COVID-19 pandemic

**Figure 5:**
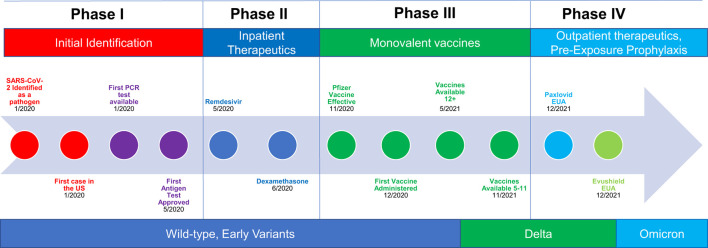
Pandemic timeline and phases: interventions, innovations, and the pathogen all experience constant, longitudinal change.

The COVID-19 pandemic can be viewed as occurring in multiple phases, each characterized by different therapeutic and preventative advancements, resource availability, pathogen infectiousness, evidence and understanding, and varying levels of feasibility, and acceptability of different mitigation policies (Figure 5). Through this lens, the first (early) phase of the pandemic in the United States lasted from approximately February, 2020 through May of 2020, and was characterized by limited understanding about the novel disease, limited access to testing, and no known effective treatments. Case fatality rates were high, as were levels of perceived fear and risk, which translated into high levels of perceived appropriateness of non-pharmaceutical interventions (NPIs). The second (late early) phase lasted from approximately May of 2020 to June of 2020 and was characterized by the identification and availability of effective inpatient therapeutics (remdesivir and dexamethasone). During Phase II, case fatality rates were lower but still relatively high compared to later periods, and access to a variety of different mitigation strategies, including testing, increased substantially. The third (middle) phase occurred from November 2020 to November 2021 and was characterized by advancements in preventative therapies, specifically vaccines with durable protection against severe disease. During Phase III, case fatality rate plummeted, fear and perceived threat fell precipitously, and the acceptability and appropriateness of many NPIs dropped substantially. The fourth (late) phase occurred from December 2021 to December 2022 and was characterized by the expansion of therapeutic options to include outpatient therapies and pre-exposure prophylaxis for those at high risk of disease despite vaccination. These advancements further lowered case fatality rates and perceived fear and therefore appropriateness of various NPIs. A fifth phase may be defined by the loss of pre-exposure prophylaxis due to pathogen evolution (13, 14). Future phases may be defined by the emergence of new variants, the development of next generation vaccines, new pharmaceuticals, or improvements in pre- and post-exposure prophylaxis options, similar to how pre-exposure prophylaxis (PREP) altered the course of the HIV epidemic (15).

### Changing resources

Availability of pandemic mitigation tools varied, impacting the feasibility, acceptability, and appropriateness of different pandemic policy responses (Table 2). Initially, NPIs, including masking, testing strategies, ventilation interventions, and distancing were the only available tools to mitigate transmission; during the very early phases, testing and personal protective equipment (PPE) were both limited resources in the United States. Subsequently, advancements in disease management and therapeutics reduced disease severity, and then development and distribution of vaccines further reduced disease severity and increased immunity in the population. Expanded access to testing and innovations in community-based surveillance methods changed the utility and delivery of this strategy over time (16).

**Table 2:** Longitudinal changes in person-based testing strategies: how implementation outcomes varied through different pandemic phases.

**Pandemic period**		**Diagnostic testing**	**Surveillance testing**	**Test-to-stay**
Early	*Key resource considerations*	*Limited availability of all tests; tests limited to those with severe disease and specific exposures*		
	Feasibility	Low	Low	Low
	Acceptability	N/a	N/a	N/a
	Appropriateness	N/a	N/a	N/a
	Cost	N/a	N/a	N/a
Pre-Vaccine	*Key resource considerations*	*Antigen testing remained in limited supply, long delays in PCR testing results*		
	Feasibility	Moderate	Variable, possible in some settings (e.g., healthcare)	N/a
	Acceptability	High	High	N/a
	Appropriateness	High	High	N/a
	Cost	Low	Low	N/a
Post-Vaccine, Pre-Omicron	*Key resource and contextual considerations*	*Both antigen testing and PCR widely available in community and healthcare settings; contact tracing feasible due to specifics of the circulating variant*		
	Feasibility	High	High	High
	Acceptability	High	Moderate	High
	Appropriateness	High	Moderate	High
	Cost	Low	High	Low
Post-Omicron	*Key resource and contextual considerations*	*More transmissible and immune-evasive variant with frequent exposures in a variety of settings. Vaccine availability to all school-aged children reduced risk of severe disease in this population*		
	Feasibility	High	High	Low
	Acceptability	High	Low	Low
	Appropriateness	High	Low	Low
	Cost	Low	High	High

Applying implementation outcomes to testing strategies at different stages in the pandemic helps to determine adoption/uptake and also public health impact. Changes in resources, feasibility, perceived acceptability and appropriateness, costs of the intervention and changes in the host-pathogen relationship determined the longtidunal public policy impacts of the same mitigation measure (testing). Categories do not apply to community-based surveillance methodologies, such as wastewater testing.

In other countries, resource access was available at different times during the pandemic. For example, in South Korea, an extensive testing program was available very shortly after identification of SARS-CoV-2, and thus the country was able to effectively leverage this strategy for early outbreak control (17–20). Testing in the US was delayed for a variety of reasons, limiting the effectiveness of a test-and-quarantine mitigation strategy. Access to PPE was also highly variable, impacting the feasibility of this approach, and therefore its potential impact.

### Implementation outcomes as determinants of NPI impact

With the exception of ventilation upgrades, most NPIs inherently require a high level of ongoing individual effort; if fidelity (or use as-intended) is not maintained, then the effectiveness of the intervention wanes. Masks, for example, only have the potential to work if people wear them (and wear them as-intended); potential policy impact is determined by adherence (21). For these reasons, NPI policy effectiveness at any point in time is necessarily determined by the degree of appropriate use as-intended within the target population or community. Use as-intended, or fidelity, in turn, is determined in part by the feasibility, acceptability, and perceived appropriateness of the intervention for the end-user at any given time. These implementation outcomes all change depending upon a variety of factors and cannot themselves be assumed to be static.

In the longer-term, policy impacts are also determined by the *sustainment* of fidelity to the intervention; because NPIs provide only short-term protection, without sustainment, their impact is to *delay*, rather than to *prevent*, infection. Fidelity, penetration, costs and sustainability are implementation outcomes that provide a means to evaluate the implementation success of interventions, treatments, policies and protocols and are distinct from traditionally measured outcomes, such as clinical or health service outcomes (11). They are also critical for considering NPI policy effectiveness as a dynamic, rather than static, entity and point to pathways to improve evidence-based public health policy recommendations during future pandemics through ongoing measurement and re-evaluation of each of these implementation outcomes.

Multiple findings from different contexts and different places in the pandemic highlight the challenges in long-term sustainment of NPI adherence and therefore the potential impact of masking policies as a pandemic control measure. For example, the Bangladesh cluster randomized controlled trial found that a bundle of implementation strategies effectively increased mask use by 29% (to 42%), and that the increase in mask use was associated with a statistically significant reduction of 10% of symptomatic SARS-CoV-2 cases in villages randomized to receive surgical masks and a non-significant reduction of 5% among villages randomized to receive cloth masks (22). An equally important finding of this large, cluster randomized controlled trial was the lack of sustainment of adherence to mask wearing over a relatively short period of time (22). Despite a nationwide masking mandate during the entire study period, a follow-up evaluation of mask use 8-12 weeks after the active intervention found that ongoing fidelity to mask use in villages randomized to receive the bundle of implementation strategies had fallen substantially. The initial 29% increase had fallen to only 10% relative to control villages. Other studies in other settings have similarly found challenges with intervention fidelity (23). Even individuals who undergo training to wear masks properly often are unaware of best practices (24). Thus, the Abaluck study and others highlight two key points about pandemic mitigation policies. First, masking policies have the potential to be beneficial, but the long-term sustainment of appropriate use or fidelity to the intervention (and therefore its impact on transmission) means that in the real world, the *public health policy impact* may change substantially as motivations, such as perceived risk and intervention fatigue, change. Recent nation-wide data provides empirical support for the condition-dependent and dynamic impacts of masking policies, highlighting the importance of incorporating implementation science principles as part of ongoing policy re-evaluation (25, 26).

The perennial challenge of sustaining fidelity to interventions that require ongoing behavioral changes (and therefore public health policy impact) is also supported by evidence about adherence to other NPI policies. A cross-sectional survey conducted in the United States about self-reported adoption of social distancing recommendations found a slow but steady decline in a variety of different settings from May to July of 2020; these findings are also corroborated by Google movements data, which demonstrated a slow but steady return to usual activity after the initial disruption (27, 28). Similarly, although school closures temporarily reduced childhood social interactions, over a relatively short time horizon and long before schools re-opened, the number of contacts increased among children, providing at least a partial explanation for the limited real-world effectiveness of this intervention (29–31). Additional data from the spring of 2020 demonstrate that movement increased most substantially among counties that lifted stay-at-home orders, but that increased community activity was also evident among counties that maintained stay-at-home policies (32). Of note, these data were collected during an early pandemic period *before* widespread immunity from natural infection and vaccination when therapeutic options were still minimal. Despite these contextual factors, fidelity to the intervention nonetheless fell, likely driven by changes in risk perception and costs associated with social distancing policies, particularly business and school closures.

Six et al. evaluated factors associated with self-reported adoption of government-recommended NPIs at different snap-shots during the first phase of the pandemic in Belgium (33). The first survey occurred during the country-wide lockdown, when cases, hospitalizations and deaths were all close to the initial peak. The second survey was collected after cases, hospitalizations and deaths had all fallen, and relaxation of mitigation measures had been announced. The third survey occurred when cases, hospitalizations, and deaths were all very low, and all mitigation measures were about to be lifted. In these different contexts, authors found that factors and perceptions associated with self-reported compliance varied. At all three data points, fear of COVID-19 severity, perceived rule appropriateness, and observing others respect the rules were positively associated with self-reported fidelity to recommended interventions. Perceptions about individual risk of exposure to COVID-19 were positively associated with increased support in the second and third surveys. Perceived rule effectiveness was positively associated with fidelity to interventions during the second survey, and measures of altruism were positively associated with self-reported fidelity to interventions during the third survey. Notably, authors also found that self-reported fidelity was *negatively* associated with trust in government. This finding diverged from a body of prior evidence suggesting that increasing trust in government is associated with increases in uptake of policy recommendations. Although the reasons for this finding about trust in the specific context of the pandemic could not be entirely delineated, authors theorized that those who were the most fearful about COVID-19, and who therefore were strongly supportive of ongoing restrictions, were also those who lost the most faith in the government response when mitigation measures were relaxed.

Findings about self-reported factors associated with appropriate use of mitigation measures align with real-world findings about policy impacts and effectiveness. Drivers of *appropriate use*, which is essential for *policy effectiveness*, and therefore *public health impact* changed over time (Table 3). Early in the pandemic, fear of COVID-19 was a major factor that drove adherence and willingness to support NPIs. Changing perceptions about disease severity – as treatments and vaccines became available and as estimates of case fatality changed – likely drove behavioral changes which, in turn, impacted policy effectiveness. Thus, Six et al.'s study also highlights the importance of updating policies to align with current contexts and public perceptions; in a system of constant and dynamic change, public health policy impacts cannot be assumed to be static. Processes for identifying key inflection points in public opinion and contextual changes to trigger policy updates are needed to improve public health policy impact.

**Table 3:** Availability of different COVID-19 mitigation measures and their acceptability, feasibility, sustainability, and potential policy adjustments as conditions and contexts changed.

	**Intervention**	**Sustainability**	**Policy adaptations**
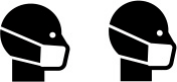	Masking mandates	Low	Shift to individual choice, change recommendations about mask type
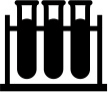	Testing programs	Variable	Based on community risk level and resources, consider non-invasive options
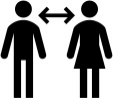	Social distancing	Very Low	Avoid in current context
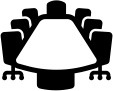	Business and Other Closures	Very Low	Avoid in current context
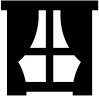	Ventilation	High to Very High	Focus on infrastructure upgrades, research
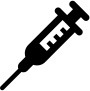	Vaccination	High to Very High	Focus on first doses and tailor boosting messaging
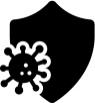	Pre-exposure Prophylaxis	High	Distribute to immunocompromised, encourage additional research

### Dynamic policy impact: implementation outcomes as determinants of real-world effectiveness

Stemming from changing contexts, evidence, and perceptions, the effectiveness of community public health measures are dynamic. Acknowledging dynamic impact implies that ongoing adaptation of public health policy recommendations as new data emerge and resource availability changes will lead to optimal evidence-based policy making and suggests a path for improving future public policy responses to infectious diseases threats.

Each of the four phases of the COVID-19 pandemic is characterized by different levels of fear, public perceptions about appropriateness, resource availability, feasibility, and differences in estimates of policy impacts (Table 3, Figure 3). The evidence basis to support NPI measures, and our understanding about how, when, and for how long they are effective also changed over time. In addition to NPI fatigue, these constantly evolving factors changed mitigation measure effectiveness via several mechanisms. Feasibility of some interventions, such as testing and contact tracing, was variable. During the early phases of the pandemic in the US, testing was not a viable prevention strategy in community settings due to limited resource availability (34); however, a test-and-quarantine strategy was successfully implemented in other countries (18). As the resource became more widely available in the US, applications of testing interventions as a mitigation measure were expanded. For example, test-to-stay programs for school settings replaced disruptive at-home quarantines in favor of in-person learning opportunities for asymptomatic students (35). However, after the emergence of the more transmissible omicron variant, contact tracing became infeasible due to the frequency of possible exposures both inside and outside of school settings, and some test-to-stay programs had to be retired or adapted (36). As noted above, longitudinal reductions in intervention fidelity were found with social distancing measures as early as the first phase of the pandemic.

Decreasing levels of use as-intended (e.g., fidelity) should be anticipated and factored into public health policy recommendations. Perceived appropriateness of different measures is also variable and dependent upon key milestones (e.g., vaccine development and distribution to high-risk populations). Widespread availability and access to vaccines decreased case fatality rates and decreased fear of COVID-19. Decreased case fatality and fear then altered the perceived appropriateness of ongoing strict mitigation policies. Alterations in perceived appropriateness led to changes in NPI uptake, which then decreased potential NPI policy impact. The lower risk of severe and fatal infections conferred by natural and vaccine-induced immunity also caused smaller absolute risk reduction associated with NPIs policies. Simultaneous with decreasing impact due to fidelity and sustainment challenges, as the duration of some of the interventions increased, most notably school and business closures, their negative impacts became increasingly apparent (37–40). Overtime, these costs altered perceived appropriateness and acceptability of these pandemic mitigation policies. Thus, multiple changes at multiple levels changed the net public health impact of NPI policies, all in the direction of decreasing potential public health impact.

Owing to a confluence of these different mechanisms, the effectiveness of NPIs is likely reduced every time they are recommended or reintroduced. Maximal potential impact occurred during the early phases of the pandemic and subsided with each subsequent wave and medical advancement. Empirical evidence from business closures suggests a potent short-term benefit with rapidly decreasing returns and increasing harms, supporting the theoretical view of longitudinal decreasing expected benefit (41).

### Sustainment versus sustainability – defining infectious disease policy goals

A core concept in implementation science is that improving uptake of an evidence-based intervention, and sustaining that increased use longitudinally, leads to improvements in clinical and public health outcomes. Embedded in this view is the concept that the effectiveness of the intervention for improving the outcome of interest is *static;* that is the effectiveness of the intervention is a *constant* value that is not inherently variable.

Responses to infectious disease threats, which always involve a dynamic host-pathogen relationship, raise the question of how public health policy goals should be defined. A traditional view of sustainability is the “fidelity approach,” which Berta et al. define as “the extent to which an intervention program follows the originally intended implementation plan and faithfully delivers the research-informed components of the intervention (42).” In the traditional view of sustainability, maintaining compliance with the originally intended evidence-based intervention is critical for achieving public health benefit. An alternate view, particularly germane to infectious diseases policy response planning, is the “adaptive approach,” which highlights the importance of the “co-evolution of the intervention” and the context. The “adaptive approach” postulates that adapting the intervention (and reducing fidelity to the original program) to better fit the context may in fact *enhance* outcomes.

Thus, rather than focusing sustainability efforts on maintaining compliance with interventions (such as masking policies), an alternate (“adaptive approach”) view is that sustainability efforts should focus on prioritizing the shifting use and form of interventions in order to maintain (or improve) public health *outcomes*. In the example of COVID-19, this translates to sustained use of interventions that prevent severe disease, long-term disability, and death, rather than focusing on sustained use of any specific intervention. Early in the pandemic, when medical countermeasures were unavailable, SARS-CoV-2 cases were highly correlated with severe disease and death. Thus, strategies that prevented cases led to reductions in mortality. However, after the availability of medical countermeasures, infections and severe outcomes became uncoupled (43–45). After this uncoupling, preventing cases had a substantially lower public health impact. Focusing on sustaining specific *interventions*, such as masking policies, therefore had a progressively decreasing impact on population health outcomes. Focusing public health policy on sustaining and improving *outcomes* is expected to have ongoing population health benefits. Notably, viewed through this lens and the “adaptive approach,” de-implementation is an inherent aspect of dynamic sustainability for infectious diseases threats, as interventions that are no longer effective should no longer be recommended or enforced.

## Maximizing public health policy impact: toward a dynamic infectious diseases public health response framework

COVID-19 is presented as a case study for considering public health policy impacts and adaption through an implementation science lens. Principles about dynamic change are inherent to the host-pathogen interaction and generalizable beyond the specifics of the public health response to the COVID-19 pandemic. Key lessons learned include the changing public health policy effectiveness of interventions as a function of conditions, contexts, and political environments and the need to consider the aims of sustainability. Rather than focusing on maintaining compliance with any specific intervention, to improve health, public health policy goals should aim to reduce severe health outcomes.

Achieving the ideal of adapting policy to sustain benefit will require re-focusing public health surveillance and evaluation methods to include consideration of implementation outcomes and changing contextual factors. Ideally, systems will be developed so that key inflection points, or phase transitions (e.g., from the early phases characterized by fear and limited resources to the later phases characterized by reduced mortality) can be measured and acted upon in real time through ongoing policy updates. Empirical evidence for the importance of novel data sources and integrating implementation outcomes into infectious diseases pandemic planning is illustrated in the modeling data from Chang et al., which demonstrated that integration of cell phone movement data improved outbreak prediction model accuracy (46). Authors also found that integration of data about compliance with social distancing policy (fidelity to the public health intervention), which varied with time, lead to persistent improvements in model prediction accuracy. This study therefore highlights the importance of integrating data about key implementation outcomes, such as fidelity, to improve infectious diseases management and public health policy.

**Figure 6:**
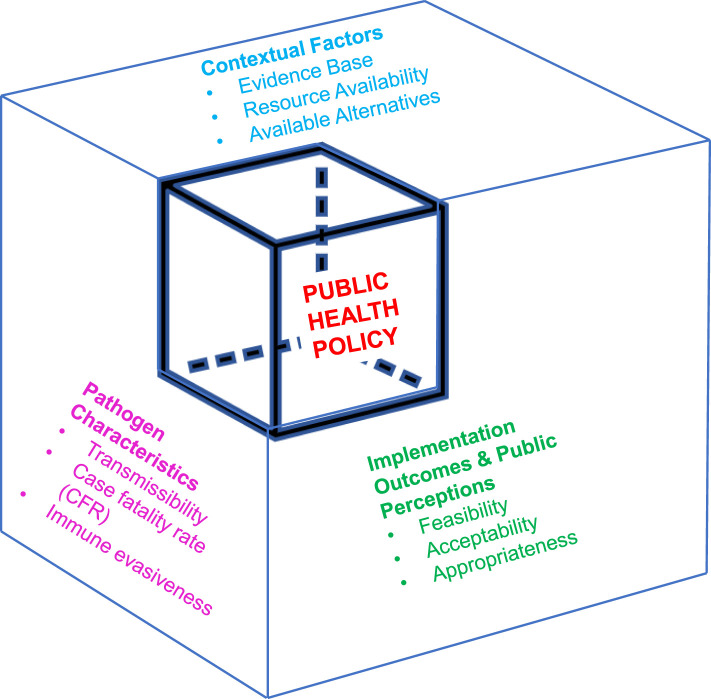
Dynamic infectious diseases public health response framework for incorporating implementation outcomes into public health policy adaptation. The dynamic infectious diseases public health response framework presents variables that impact public health policy effectiveness. Optimizing the effectiveness of public health policy necessitates ongoing evaluation of each of these different variables, with updates as they change.

The *Dynamic Infectious Diseases Public Health Response Framework* is a model for evaluating ongoing public health policy impact in the context of a constantly changing and evolving system (Figures 3, 6). Concepts are grounded in implementation science theory and are a direct extension of the Dynamic Sustainability Framework (10). Key considerations are generalizable to many infectious diseases threats and associated mitigation measures and prevention responses.

Focusing on maximizing *public health outcomes* has additional implications. For populations that are high-risk of severe disease despite vaccination (or for other reasons, depending upon the specific infectious disease in question) (47–49), different policy recommendations may be needed to achieve public health goals. For example, mitigation measures designed to prevent any COVID-19 case applied in skilled nursing facilities, dialysis centers, and chemotherapy units are expected to have a more substantial direct public health impact than when those same mitigation measures are applied to lower-risk populations, such as interventions implemented in elementary and secondary school settings. Acknowledgment of differential population risk was one of the reasons masking requirements were maintained in hospital settings longer than in the community (50). Thus, implementation of a Dynamic Infectious Diseases Public Health response requires measurement of population risk and adaptation of policy to match level of risk. In this setting, risk should be defined broadly, and harms and costs of the intervention should be included when crafting public health policy.

Achieving future improvements in real-time infectious disease policy responses will require major infrastructure investments to collect the data necessary to inform ongoing public policy decisions. Traditional surveillance systems focus on measuring cases via reporting from state and local health departments. These traditional systems do not have the infrastructure or linkage to data elements that would facilitate evaluation and integration of implementation outcomes. Novel mechanisms for gathering and interpreting data in near-real time are needed; practically, this will likely include a national data repository, technologic advancements in data cleaning and real-time analysis, and integration of non-traditional sources of information, such as social media, for ongoing assessment of public perceptions about different public health policies. For example, measuring trends about discussion of masking policies and school closures on Twitter and other social media sites likely would have provided valuable insight into changing acceptability of these public health recommendations, and allowed for public health policy makers to integrate perceptions of end-users into ongoing policy updates. Similarly, early in the pandemic, ongoing discussions of lack of access to testing may have served as a signal to invest more heavily in this strategy.

Inherent in the Dynamic Infectious Diseases Public Health Framework is the concept that as implementation outcomes evolve longitudinally, so does the effectiveness of public health policy responses (Figure 1). Thus, as a direct result of constantly evolving conditions, risk of the setting and to the individual, public perceptions, and political contexts, public health policy needs constant revision and evolution to maintain the same societal benefits (7).

Challenges with sustainment of behavior change to control disease spread are not specific to COVID-19. Recommendations for use of barrier methods to prevent transmission of sexually transmitted infections, particularly HIV infection, and to reduce the number of sexual contacts, were eventually abandoned in favor of a more harm-reduction focused approach (51), as early abstinence-based public health recommendations were found to be unacceptable to many and therefore less effective than other approaches, such as treatment and PrEP (52–55). In the summer of 2022, when the Mpox outbreak occurred, public health recommendations that focused on reducing sexual contacts were heavily criticized, due in part to lack of acceptability and perceived appropriateness of the intervention among recipients of the public health campaign (56–59).

## The dynamic infectious diseases public health response: an extension of the real-time, learning health system approach

The ideal of the Learning Health System is that data generated in real time can be leveraged to advance our scientific understanding of a problem and that advancement in our understanding can be leveraged to improve care delivery (6). Inherent in this model is the idea that “the evidence” is constantly changing and evolving, and that a variety of data elements are needed to realize potential benefits of this approach (60). Particularly when applied to the context of public health programs, data elements include not only traditional data elements but also other factors that impact real-world effectiveness – cultures, believes, attitudes and contexts. Integration of these other elements into policy evaluation is critical for achieving and sustaining public health benefit.

Adaptation to changing conditions and contexts is the best way to incorporate evidence into policy changes but achieving the ideal synergy of specific policy recommendation to context and public perception requires substantial advancements and investment in data collection, analysis, and implementation. Substantial changes in contexts, evidence and phases need to be identified in near-real time, so that public health policy can be aligned with the current conditions. The more heavily reliant an intervention is on day-to-day, person-level appropriate use for effectiveness, the more strongly the duration of the intervention should be considered in weighing recommendations. Ideally, evaluations of the effectiveness of individual-level interventions should be ongoing, with constant reassessments of whether the intervention is continuing to achieve the desired outcome and immediate policy change once the intervention is no longer effective or found to be harmful. Costs and harms of an intervention should be evaluated in parallel with benefits and on an ongoing basis. Improvements in real-time data analytics are likely to result in earlier policy de-implementation as human behaviors and expectations are inherently integrated into public health policy making. Once the balance tips away from benefit and toward harm, the policy should be de-implemented, with clear and open communication about the emerging evidence and rationale for the change.

Recognizing the inherent limitations of observational data, advancements in data management and rapid data analysis are needed for the next pandemic to enhance our understanding about real-time policy effectiveness. Given the role of perceived appropriateness of policy in driving compliance—and therefore effectiveness—improvements should also include mechanisms to assess and integrate public feedback into public health policy and messaging. Data about the feasibility, acceptability, and costs of different interventions, such as social distancing policies and business and school closures should be collected by public health officials and state and local governments from publicly available sources, such as social media platforms and google movements as well as directly from interested parties and individuals impacted by specific policy interventions. These data elements should then be integrated into planning and policy responses. Data about ongoing use could be used to inform whether policy effectiveness is likely to have changed due to changing conditions and public perceptions. Potential harms of interventions should be prospectively measured and incorporated into assessments of the ongoing acceptability and appropriateness of the public health policy. These data elements could then be mapped to quantitative data and used to tailor public health policy recommendations based on input from those most impacted and ongoing expected benefits and harms. An example application of integrating implementation outcomes into public policy design is presented in Table 3, which ranks NPIs according to feasibility and acceptability. In the future, information about implementation outcomes and public perceptions could be integrated with evidence about relative policy effectiveness to adapt public health policy recommendations to align with current conditions to sustain benefits that are informed by factors that are important to the public.

## Practical considerations

Achieving this ideal – rapid data collection and analysis to inform on-the-ground policy recommendations will require substantial investments in national informatics infrastructure to achieve a public health ‘Learning Health System' (6, 7, 61). Leveraging advancements in artificial intelligence to link and analyze novel data sources, such as social media commentary, to assess ongoing public perceptions, feasibility, and acceptability may help in the future to realize these ideals. Setting up systems where data are assembled, analyzed and interpreted, leading to knowledge of the needed interventions and how to manage them, and then tracking data to understand practices changes in real-time are learning health system steps required for the real-world application of the Dynamic Infectious Diseases Public Health Framework (60).

A practical tool to assist policy makers in applying the framework is presented in Supplementary material 1. The practical tool with pre-populated responses to key determinants of public health policy impacts directs iterative assessments of key implementation outcomes and contextual factors that can be evaluated in real-time to inform and adapt public health policy. It is designed to facilitate measurements and incorporation of implementation outcomes, such as feasibility, appropriateness and costs to inform and adapt decision making, which may include policy adaptation, policy de-implementation, or determination that additional information is needed. If additional information is needed, the tool can help to identify knowledge gaps and direct scientific investigation to close these gaps. Integration of novel information sources, such as public input from social media platforms, is encouraged as part of ongoing assessments of public perceptions about policy appropriateness and harms. Key population-specific factors that may impact decision-making, such as risk of disease, are also included to inform policy development and adaptation. Optimizing the effectiveness of public health policy necessitates pre-planned, ongoing evaluation of each of these different variables, with updates as changes are identified.

**Figure 7:**
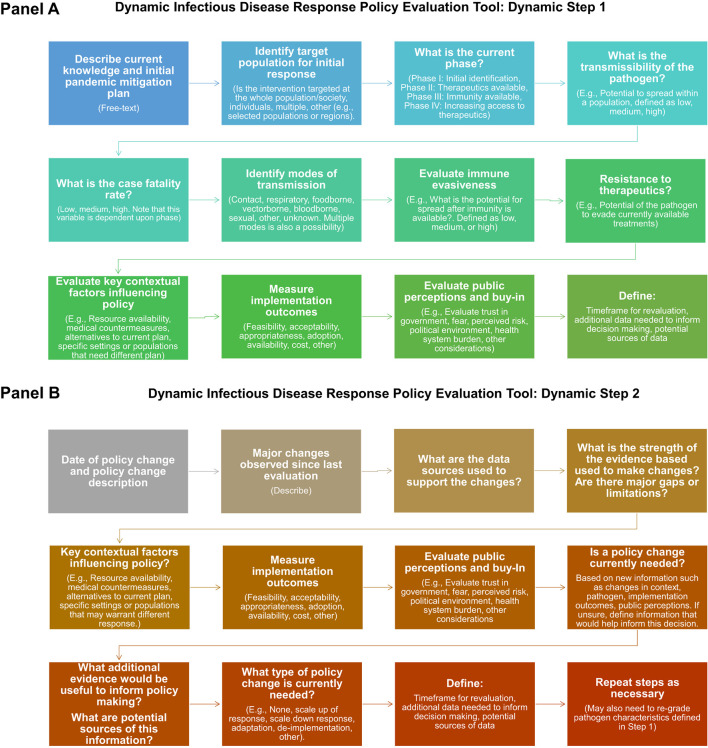
Steps in the dynamic infectious diseases public health response framework practical took. **(Panel A)** depicts the questions evaluated during Step 1 of the policy making process, which includes initial plan, evaluation pathogen characteristics, current evidence, implementation outcomes, and public perceptions. At the end of step 1, users define the timeframe for re-evaluation and key information gaps and potential sources of data to inform ongoing policy evaluation and adaptation. **(Panel B)** depicts the questions evaluated during step 2, which should be repeated as often as is necessary to achieve public health policy goals.

A flow chart to direct application and use of the tool is presented in Figures 7A, B. Figure 7A depicts the first step of the dynamic response, which includes consideration of pathogen characteristics, key contextual factors including the current evidence base and potential alternatives to the current strategy, assessment of implementation outcomes (defined in Table 1), and public perceptions. These steps are delineated to assist with policy making and to identify key challenges and evidence gaps to inform subsequent decision-making. At the end of dynamic step 1, users should define the timeframe for re-evaluation. Dynamic step 2 is depicted in Panel B. Step 2 defines key factors that may merit a policy change and directs ongoing planning, including collection of new evidence. Step 2 is designed to be repeated as many times as is necessary for the duration of the response.

## Summary and conclusions

A refrain throughout the pandemic has been “the science has changed!” Public health policy making is complex – and the message that impacts of public health policies are dynamic is a difficult one to convey. While knowledge and evidence have evolved and expanded, fundamental scientific principles have not. Diagnostics, therapeutics, preventative interventions, and viral variants did change, as did public perceptions, tolerances, and behaviors. In future policy responses and policy messaging, uncertainty must be acknowledged and embraced. Public health officials should also be upfront that change in policy is an expected outcome of the most evidence-based practice, as we learn more, the context, conditions, and evidence change, and even the goals of the public health response evolve. Both implementation and de-implementation plans should be incorporated into planning.

The Dynamic Infectious Diseases Public Health Response Framework is presented through the lens of the COVID-19 pandemic response but is broadly applicable to public health interventions that include complex and ever-evolving host-pathogen interactions. Consideration of implementation outcomes, in addition to more traditional measures of clinical effectiveness, may help to improve evaluations of public health programs and impact and to facilitate matching policy recommendations with evolving contexts. Public health policy goals, feasibility, costs, and perceived acceptability and appropriateness change as the context, evidence, and resources change, highlighting the importance of viewing the impact and effectiveness of public health policies and impacts as dynamic elements of a larger constantly evolving and changing system. These implementation outcomes are determinants of ultimate public health policy impact.

Public health policy and pandemic responses are not just about the evidence– or just about the evidence at one moment in time. This is particularly true for the management and control of infectious diseases, which always involve a dynamic interplay between the host and the pathogen. Real-time decision making requires sensitivity to conditions on the ground and adaptation of intervention at all levels as implementation outcomes, such as acceptability, appropriateness, and fidelity change and as contexts evolve. When asking about the public health effectiveness and impact of non-pharmaceutical interventions, the focus should be on *when, how*, and *for how long* they can achieve public health impact – definitive statements such as “masks work” or “masks don't work” fail to capture how interventions work in real world settings and contexts.

Static effectiveness estimates cannot be assumed in a constantly changing system. Policy impacts are dynamic and need to be recognized and evaluated as such. Just as NPI policy should change, our public health infrastructure needs to adapt to maintain effectiveness in the background of constant change and to maintain relevance and benefit to the end-user of these policy recommendations: the public.

## Author contributions

All authors contributed to concept review and analysis, drafting, and editing of the manuscript. In addition, all authors met with the graphic designer and assisted in the design and revision of the figures presented in the manuscript.
